# Spermatotoxic Effects of Single-Walled and Multi-Walled Carbon Nanotubes on Male Mice

**DOI:** 10.3389/fvets.2020.591558

**Published:** 2020-12-17

**Authors:** Omid Farshad, Reza Heidari, Mohammad Javad Zamiri, Socorro Retana-Márquez, Meghdad Khalili, Melika Ebrahimi, Akram Jamshidzadeh, Mohammad Mehdi Ommati

**Affiliations:** ^1^Pharmaceutical Sciences Research Center, Shiraz University of Medical Sciences, Shiraz, Iran; ^2^Department of Animal Science, Shiraz University, Shiraz, Iran; ^3^Department of Reproductive Biology, Universidad Autónoma Metropolitana-Iztapalapa, Mexico, Mexico; ^4^Department of Pharmacology and Toxicology, School of Pharmacy, Shiraz University of Medical Sciences, Shiraz, Iran; ^5^College of Life Sciences, Shanxi Agricultural University, Taigu, China

**Keywords:** environmental toxicology, nanomaterials, nanomedicine, oxidative stress, toxicity, sperm

## Abstract

Carbon-based nanomaterials possess a remarkably high potential for biomedical applications due to their physical properties; however, their detrimental effects on reproduction are also concerned. Several reports indicate the toxicity of carbon nanotubes (CNT); nevertheless, their impact on intracellular organelles in the male reproductive organs has not been fully elucidated. Herein, we report on the reprotoxicity of single-walled (SWCNT) and multi-walled carbon nanotubes (MWCN) on several intracellular events and histological criteria in pubertal male BALB/c mice orally treated with 0, 10, and 50 mg/kg/day doses for 5 weeks. Biomarkers of oxidative stress and mitochondrial functionality, histopathological alterations, and epididymal sperm characteristics were determined. Oral administration of CNTs at 10 and 50 mg/kg evoked a significant decrement in weight coefficient, sperm viability and motility, hypo-osmotic swelling (HOS) test, sperm count, mitochondrial dehydrogenase activity, ATP content, total antioxidant capacity, and GSH/GSSH ratio in the testis and epididymal spermatozoa. On the other hand, percent abnormal sperm, testicular and sperm TBARS contents, protein carbonylation, ROS formation, oxidized glutathione level, and sperm mitochondrial depolarization were considerably increased. Significant histopathological and stereological alterations in the testis occurred in the groups challenged with CNTs. The current findings indicated that oxidative stress and mitochondrial impairment might substantially impact CNTs-induced reproductive system injury and sperm toxicity. The results can also be used to establish environmental standards for CNT consumption by mammals, produce new chemicals for controlling the rodent populations, and develop therapeutic approaches against CNTs-associated reproductive anomalies in the males exposed daily to these nanoparticles.

**Graphical Abstract:**
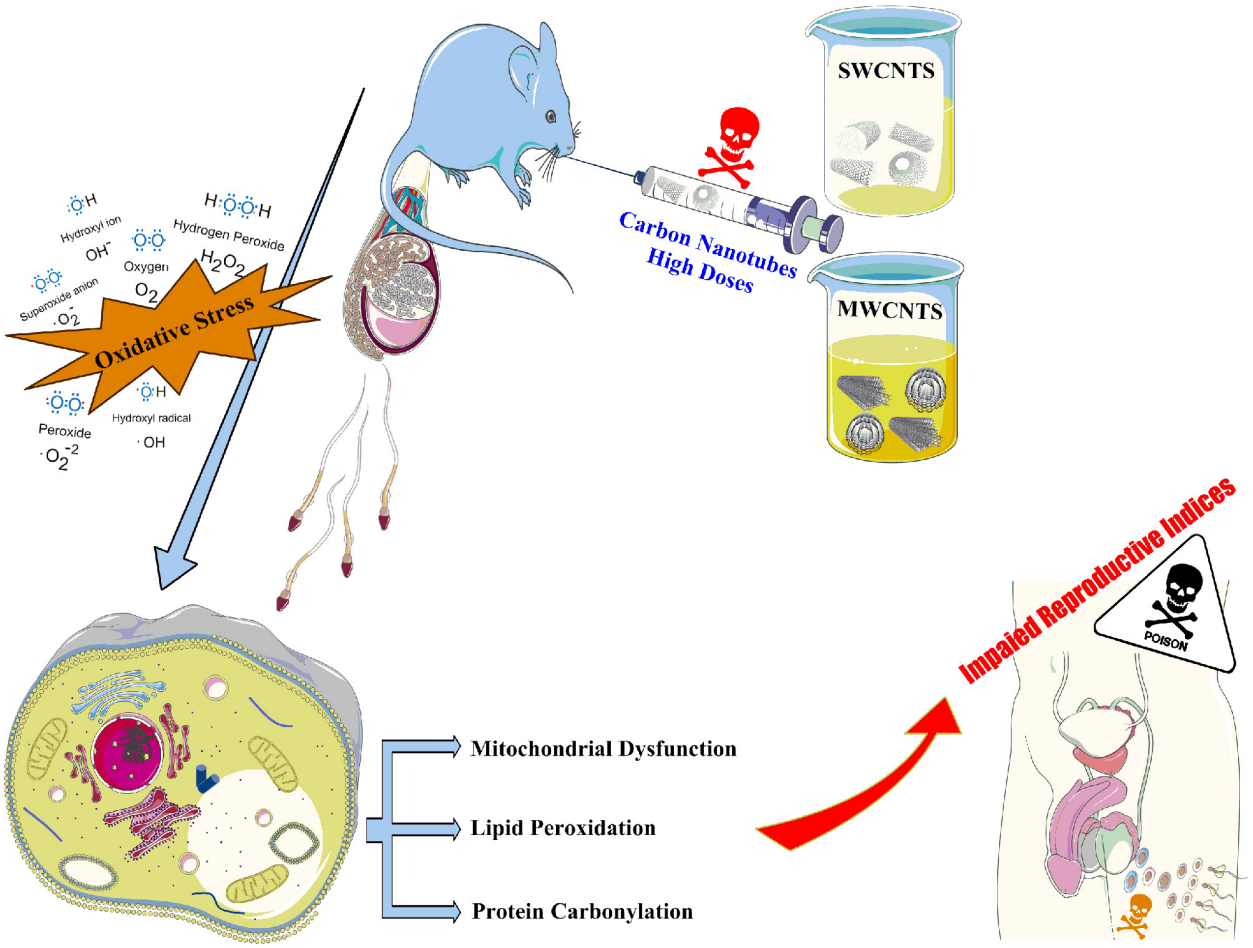
Oxidative stress-induced mitochondrial impairment was noticeably involved in CNTs-induced reproductive system injury and Spermatotoxicity.

## Highlights

- Xenobiotics-induced reproductive toxicity and infertility is a big challenge.
- Male mice were exposed to 10 and 50 mg/kg of SWCNTs and MWCNTs for five weeks.
- Biomarkers of oxidative stress were considerably changed in the testis of CNTs-treated animals.
- The CNTs not only caused considerable deleterious effects on male gametes but also altered the mitochondrial indices of functionality.
- These data indicate CNTs induced reproductive system injury and spermotoxicity through oxidative stress and mitochondrial impairment.

## Introduction

Carbon nanotubes (CNTs) belong to the new superfamily of nanomaterials, with remarkable physical properties (i.e., high strength and stiffness as well as low density and great thermal conductivity) (1–4). These impressive properties have created a substantial industrial interest and application for these light-weight high strength tubes (4, 5), culminating in the design and execution of many experimental and theoretical studies. The biomedical usage of CNTs is promising (6–8); however, their potential toxic risks remain still a concern. Two major structural forms of CNTs are single-walled carbon nanotube (SWCNT) bundles and multi-walled carbon nanotubes (MWCNT). Availability in the market and studies on CNTs have continuously increased (9). These compounds are widely used in the pharmaceutical industry and as drug carriers. Acute or chronic exposure to CNTs has been associated with disorders in the brain (10), heart (11), liver (11), intestine (12), lung (13), gills (14), kidney (15), spleen (16), central nervous system (17), immune system (18), reproductive organs, and neuroendocrine system (9, 19). Although the consumption of CNTs in health-related aspects is still to be evaluated quantitatively, as the exposure risk of employees and consumers increases, qualitative assessment of the designed stable suspensions containing various forms of CNTs, is essential for safe usage in medicine.

It is well-known that there is a close relation between reproduction and developmental toxicity. Reprotoxicity is one of the crucial parts of chemicals risk assessments by evaluating the internal organs and endocrine system (i.e., hypothalamus-pituitary-gonad-sperm axis, HPG-S) (20, 21). Additionally, there is a need to clarify the reproductive and developmental health effects of CNTs (18). This area of male reproductive nanotoxicology has received limited attention. Hence, the current study aimed to evaluate the adverse effects of CNTs on the male reproductive system and clarify the possible mechanisms of their toxicity.

It has been shown that upon pulmonary exposure to CNTs, inflammatory intermediaries are released and transferred to the circulatory system. Hence, systematic inflammation could occur (22). Hougaard et al. have been reported that systematic inflammation could interfere with the reproductive indices in females (9). Therefore, further studies are needed to assess the harmful effects of inflammation-triggered mediators on the HPG-S axis of paternal generation or F1- male offspring whose parents and/or themselves received various doses of these nanoparticles.

Many factors could negatively affect reproductive functions (23). Oxidative stress (OS), mitochondrial impairment, and their subsequent complications seem to play crucial roles in the xenobiotics- induced reprotoxicity and consequent infertility (20, 21, 24–30). High sensitivity to OS, excessive reactive oxygen species (ROS) levels, and thiobarbituric acid reactive substances (TBARS) content could dramatically affect the functions of the reproductive organs (20, 21, 26–34). For instance, oxidative injury and DNA damage studies indicated that high doses of MWCNT could cause DNA damage in murine sperm (35). It has been reported that CNTs could accumulate in different organs (36, 37). The male reproductive system in humans is well-known to be at risk of various kinds of xenobiotics (38). The precise mechanism of xenobiotics-induced reproductive toxicity is complicated, but OS is one of the crucial causes (39, 40). Many investigations ascribed OS as the primary mechanism of CNTs-induced cytotoxicity and organ damage (41–43). Hence, several OS markers have been evaluated in the current study to enlighten the mechanism of CNTs reproductive toxicity.

CNTs have been shown to pass through the blood-testis barrier (BTB), blood-brain barrier (BBB), and hemato-testicular barrier (HTB) (44, 45). Such intracellular transfers can cause CNT accumulation in the testis and raise concerns about the offspring's quality. For instance, Bai et al. (45) found that soluble CNTs accumulated in the testes, generated OS, and decreased the seminiferous epithelium's thickness after about 2 weeks of treatment, even though they reported that this anomaly was repaired within 60–90 days. The BTB has a crucial role in protecting the testis against xenobiotics (46–48). Meanwhile, several xenobiotics or diseases disrupted the BTB and BBB causing anomalies in the reproductive and non-reproductive systems (48–50). Hence, the effects of CNTs on the tight junctional structure and related gene/protein expression (such as Occluding, Claudin, ZO-1, and ZO-2) in these barriers are essential in investigations on CNT-related reprotoxicity.

Cellular energy metabolism is pivotal in sperm forward motility (24, 25, 51), and there is ample evidence showing that xenobiotics could alter mitochondrial functionality and induce severe OS in different reproductive tissues (20, 21, 27–30). In this regard, Xu et al. (52) reported that MWCNT caused severe damages to mitochondrial DNA in spermatocytes. They also showed a down-regulation of mitochondria-related gene expression, a decrement in oxygen consumption rate, and decreased cellular ATP level in a mouse spermatocyte cell line (GC-2spd) exposed to CNTs, even at the nonlethal dose (0.5 μg/mL). Finally, they concluded that this organelle accumulated MWCNT. These findings underscore the need to assess the effects of CNTs on reproductive health and development of the HPG-S axis in the offspring (F1–F3) whose parents and themselves have been exposed to these nanoscale elements. The present investigation aimed at determining the adverse effects of MWCNT and SWCNT on the male reproductive system in mice. Moreover, the potential mechanism of CNTs-induced reproductive toxicity was evaluated by assessing oxidative stress and mitochondrial parameters.

## Materials and Methods

### Chemicals

2′,7′ Dichlorofluorescein diacetate (DCFH-DA), 3-(N-morpholino) propane sulfonic acid (MOPS), 3-[4,5dimethylthiazol-2-yl]-2,5-diphenyltetrazolium bromide (MTT), bovine serum albumin (BSA), dimethyl sulfoxide (DMSO), thiobarbituric acid (TBA), glutathione (GSH), malondialdehyde (MDA), eosin, Coomassie brilliant blue, nigrosin, 2, 4-dinitrofluorobenzene (DNFB), dinitrophenylhydrazine (DNPH), sucrose, KCl, NaCl, dithiothreitol (DTT), Na_2_HPO_4_, MgCl_2_, Rhodamine 123 (Rh 123), ethylenediaminetetraacetic acid (EDTA), were purchased from Sigma Chemical Co. (St. Louis, MO, USA). SWCNT and MWCNT were prepared from US Research nanomaterials Inc. (Houston, TX 77084, USA; Please refer to the Supplementary Material for more information). Trichloroacetic acid (TCA) and hydroxymethyl aminomethane hydrochloride (Tris-HCl) were purchased from Merck (Darmstadt, Germany). The salts for preparing buffer solutions were of the analytical grade and obtained from Merck (Darmstadt, Germany).

### CNTs Properties, Preparation, and Stability Assessment

The single-walled carbon nanotube (SWCNT; > 95%, OD: 1–2 nm, Stock #: US4112, CAS #:99685-96-8) and multi-walled carbon nanotube (MWCNT; > 95%, OD: 20–30 nm, Stock #: US4310, CAS #:99685-96-8) were purchased from from the US Research Nanomaterials Inc. (Houston, TX 77084, USA). Due to a lack of purification or sieving technique, the required amounts of SWCNT and MWCNT (250 mg) were dissolved in 100 mL distilled water (DW), and the animals were treated as described in section Animals and Treatments.

For stability assessment, SWCNT or MWCNT (1 mg) was transferred into a tube containing 10 mL poly-ethylene glycol 400 (10% v: v) and 5 mL DW, and the tube vortexed for 5 min. After sonication for 60 min, the absorbance (λ = 880 nm) was read using an EPOCH® plate reader (BioTek® instruments, Highland Park, USA). All steps were performed at 4°C.

### Animals and Treatments

Forty pubertal male BALB/c mice, 3-week-old at the commencement of the study (weighing ~15 g), were purchased from the Experimental and Comparative Medicine Research Center of Shiraz University of Medical Sciences, Shiraz, Iran. The mice were randomly assigned to five treatment groups (*n* = 8 per group) and allowed a 7-day adaption period before applying the treatments as a daily gavage for the duration of a complete spermatogenic cycle (35 days) in BALB/c mice. The treatments were: (A) Control (vehicle-treated); (B) 10 mg/kg/day MWCNT; (C) 50 mg/kg/day MWCNT; (D) 10 mg/kg/day SWCNT; and (E) 50 mg/kg/day SWCNT. The CNTs doses used in the current study were selected based on previous investigations in this field (53–55).

The experimental animals were kept in an animal house at the Pharmaceutical Research Center of Shiraz Medical University (four mice per cage), having free access to commercial rodent pellets (Behparvar®, Tehran, Iran) and tap water. The environmental conditions were: 12 h:12 h light-dark ratio, 19–23°C temperature, 50–70%, relative humidity, and an air exchange rate of ≥15 times/h. Animal handling and experimental procedures were approved by the Experimental Animal Welfare and Ethics Committee of Shiraz University of Medical Sciences, Shiraz, Iran (Agreement #: 95-01-36-11290).

### Sample Collection

On day 36, the mice were euthanized (Thiopental, 70 mg/kg, i.p.), and the epididymides and testes were sampled and weighed. The left testis was fixed in 10% formalin for histopathological evaluations. Total antioxidant capacity, lipid peroxidation, reactive oxygen species (ROS) production, protein carbonylation, and glutathione contents were determined in the right testis. Sperm suspension was prepared from the cauda epididymis of the left testis.

### Male Reproductive Organ Coefficient

The weighted index (WI) for each reproductive organ was determined as:

WI = [wet weight of organ (g)/body weight (g)] × 100.

### Sperm Quality Evaluation

Epididymal sperm count and progressive sperm motility were determined as previously described (26, 32). Briefly, epididymal sperm suspension was prepared by mincing the cauda epididymis in warm phosphate-buffered saline (PBS; 35°C; pH = 7.4). Sperm forward motility was determined by transferring a drop of the epididymal sperm suspension on a glass slide covered with a coverslip and observing the spermatozoa under a Zeiss (Jena, Germany) compound light microscope (400 × magnification) equipped with a hot-stage (35°C). Sperm concentration was measured by transferring a portion of diluted epididymal fluid (10 μL) onto a Neubauer chamber and observing the cells under a light microscope (200 × magnification).

The hypo-osmotic swelling (HOS) test, as a valid index of membrane integrity, was performed by mixing 10 μL of sperm suspension with 50 μL of hypo-osmotic solution (50-mOsm NaCl) for 10 min at 37°C and calculating the percentages of spermatozoa (in at last 200 sperm per slide) with a swollen “bubble” around the curled flagellum, using light microscopy (1,000 × magnification) (31, 56). Sperm abnormality and viability were measured in duplicate (200 sperm per sample) after eosin-nigrosin staining (20, 31). Spermatozoa with protoplasmic droplets, ab-axial tails, malformed heads, double tails, coiled tails, bent tails, and without tail or head were recorded as abnormal under a phase-contrast microscope (Olympus BX41; Olympus Optical Co. Ltd, Japan) (28, 57).

### Oxidative Stress Parameters in the Testis and Epididymal Sperm

#### Testicular and Sperm Reactive Oxygen Species (ROS)

Testicular and sperm ROS levels were measured using the fluorescent probe dichlorofluorescein diacetate (DCFH-DA) (58, 59). Briefly, DCFH-DA was added (10 μM final concentration) to sperm or homogenized testicular samples (1 mg protein/mL). Samples were incubated for 30 min at 35°C in the dark, DCF fluorescence intensity was recorded using a FLUOstar Omega® multifunctional microplate reader (BMG LABTECH, Germany) (λ_excitation_ = 485 nm and λ_emission_ = 525 nm) (57, 59).

#### Testicular and Sperm Lipid Peroxidation

As an index of lipid peroxidation, thiobarbituric acid reactive substances (TBARS) were measured in the testis and isolated gametes. A sample (500 mg) of testicular homogenate (10% w/v in KCl, 1.15% w: v) or epididymal suspension (containing 10^6^ sperm/mL) was added to a reaction mixture consisting of 1 mL thiobarbituric acid (0.375%, w: v) and 3 mL phosphoric acid (1% w/v, pH = 2) (28, 60). The mixture was incubated at 100°C for 45 min. Afterward, n-butanol (2 mL) was added to the mixture and thoroughly mixed. Finally, the specimens were centrifuged (10,000 × *g* for 5 min), and the absorbance of the developed color in the n-butanol phase was recorded at λ = 532 nm by an Ultrospec 2000®UV spectrophotometer (Scinteck Instruments, United States) (61).

#### Testicular and Sperm Glutathione Content

The reduced (GSH) and oxidized (GSSG) glutathione contents were measured by the HPLC analysis of the deproteinized samples (TCA 50% w: v). The samples were derivatized with iodoacetic acid and fluoro-2,4-dinitrobenzene, using an NH_2_ column (Bischoff chromatography, Leonberg, Germany, 25 cm), at a flow rate of 1 mL/min (62). Buffer A (water: methanol; 1:4 v: v) and buffer B (acetate buffer: methanol; 1:4 v: v) were used as the mobile phases. A gradient method with a steady increase of buffer B to 95% in 20 min was used (29, 62), where the nanomolar levels of GSH and GSSG can be determined (62); GSSG and GSH were used as external standards. Specimens of testicular tissue (200 mg) and spermatozoa (10^6^ /mL) were homogenized in 250 mM Tris-HCl buffer (pH = 7.4) at 4°C. Afterward, 500 μL of TCA (50% w:v, 4°C) were added to each homogenized sample. The specimens were mixed gently and centrifuged (15,000 g) for 15 min at 4°C. A mixture of NaOH and NaHCO_3_ (2 M:2 M; 400 μL) was slowly added to 1 mL supernatant, until the gas production was subsided. Then, iodoacetic acid (100 μL, 1.5% w:v in water) was added, and samples were incubated for 60 min at 4°C in the dark. After incubation, 500 μL of DNFB (1.5% w:v in absolute ethanol) were added and incubated in the dark for 48 h at 25°C. Finally, samples (25 μL) were injected into the HPLC system, and the UV detector was fixed at λ = 252 nm (62, 63).

#### Ferric Reducing Antioxidant Power (FRAP) in Testicular Tissue

The FRAP assay evaluates any alteration in the absorbance at λ = 593 nm due to the formation of a blue-colored Fe^2+^-tripyridyltriazine from the colorless oxidized Fe^3+^ form by the action of electron-donating antioxidants (28, 64). Briefly, 10 parts of 300 mmol/L acetate buffer (pH = 3.6) with 1 part of 10 mmol/L TPTZ (2, 4, 6-tripyridyl-s-triazine) in 40 mmol/L hydrochloric acid and 1 part of 20 mmol/L ferric chloride were used to prepare the working FRAP solution which was prepared freshly on the day of the experiment. Testicular samples were homogenized in 0.25 M Tris-HCl buffer (pH = 7.4), containing 0.2 M sucrose and 5 mM dithiothreitol (DTT), at 4°C (65). Afterward, 1.5 mL FRAP reagent and 150 μL deionized water were added to 50 μL of the homogenized tissue and incubated for 5 min at 37°C. The resultant blue color intensity was measured at λ = 593 nm using an Ultrospec2000® spectrophotometer (Scinteck Instruments, United States) (66). Data were standardized by using the sample protein content (67).

#### Testicular Protein Carbonylation

A spectrophotometric technique was used to measure the oxidative damage in proteins by determining carbonyl groups based on their reaction with DNPH (68). Briefly, testicular tissues were homogenized in 0.25 M Tris-HCl buffer (pH = 7.4). Then, 100 μL of TCA (20% w:v, 4°C) were added to 1 mL of the tissue homogenate and centrifuged (700 *g*, 15 min). Afterward, 500 μL of 10 mM DNPH (dissolved in 2 N HCl) was added to the supernatant and incubated for 60 min in the dark at 20°C (vortexing every 10 min). Then, 0.1 mL of TCA (20% w: v) was added. The tubes were centrifuged (12,000 g for 5 min), the supernatant discarded, and the pellet washed three times with 1 mL ethanol: ethyl acetate (1:1 v: v). The precipitate was re-dissolved in 600 μL of guanidine solution (6 M, with 20 mM potassium phosphate, adjusted to pH = 2.3 with trifluoroacetic acid) for 15 min at 37°C. Finally, the absorbance (λ = 370 nm) was measured using an EPOCH plate reader (BioTek® instruments, Highland Park, USA) (68).

### Sperm Mitochondrial Indices

#### Mitochondrial Dehydrogenase Activity

Mitochondrial dehydrogenase activity was measured using the MTT solution (28, 57). Briefly, sperm specimens (1 mg protein/mL) were incubated with 40 μL of MTT solution (5 mg/mL, 37°C, 30 min). The purple formazan crystals, as reaction products, were dissolved in 1 mL of DMSO, and the optical density (OD) of the reaction products was determined at λ = 570 nm using an EPOCH plate reader (BioTek Instruments, Highland Park, USA) (69, 70).

#### Sperm Mitochondrial Membrane Potential

Rhodamine-123 (Rhd-123) was used to determine sperm mitochondrial membrane potential (58, 70). Samples of sperm suspension (1 mg protein/mL) were incubated with 10 μM of Rhd-123 for 30 min at 35°C in darkness, centrifuged (10,000 *g*, 4°C). The fluorescence intensity of the supernatant was measured (λ _excitation_= 485 nm and λ _emission_ = 525 nm) using a FLUOstar Omega® multifunctional microplate reader (BMG LABTECH, Germany) (58, 70).

### Testicular Histopathology and Stereology

Testicular samples already were fixed in buffered formalin solution (0.4% NaH_2_PO_4_, 0.64% Na_2_HPO_4_, and 10% formaldehyde in distilled water; pH = 7.4), were rinsed with running tap water for at least seven h and then dehydrated in graded alcohol, cleared in xylene, and embedded in paraffin. The paraffin-embedded specimens were cut into 5-μm sections (Leica Rotary Microtome RM2255, Buffalo Grove, IL) and stained (H&E staining). Histopathological evaluations were blindly performed by a pathologist using a light microscope (Olympus BX41; Olympus Optical Co. Ltd, Japan). For testis tubular desquamation, the STs were evaluated for the existence of complete spermatogenesis and focal or diffuse atrophy or tubular degeneration, depending on the number of affected tubules. Early intratubular desquamation was graded as mild (+), moderate (++), or severe (+++) (71, 72). Multinucleated cells within the tubular lumen or among spermatogenic cells were recorded (71, 72). The spermatogenic index was recorded based on the ST number ratio containing male gametes to the empty tubules (71, 72). The stereology indices, including the total, cellular, and lumen diameter of STs, as well as the number of STs and spermatogenesis index (by monitoring the spermatogonia, spermatocytes, and spermatids), were measured according to our previous investigations (20).

### Fertility Rate (FR)

The FR index was evaluated by mating each exposed mature male with two mature virgin females. The mating success was confirmed the following morning by checking for male gamete attendance in the vaginal smear. Any male who fertilized at least one female was considered as a fertile male. Copulated female mice were permitted to keep their pregnancy, and finally, the neonate number (litter size) was documented.

### Statistical Analysis

Data were expressed as mean ± SEM. The normality test was applied to data. Statistical analysis was performed according to the one-way analysis of variance (ANOVA), followed by mean comparisons using Tukey's multiple comparison test as the *post hoc* test. The significance level was set at *P* ≤ 0.05 (SPSS software, Version 19, IBM Corporation, New York, U.S.A.).

## Results

### Bodyweight Gain and Male Reproductive Organ Weight

**Figure 1:**
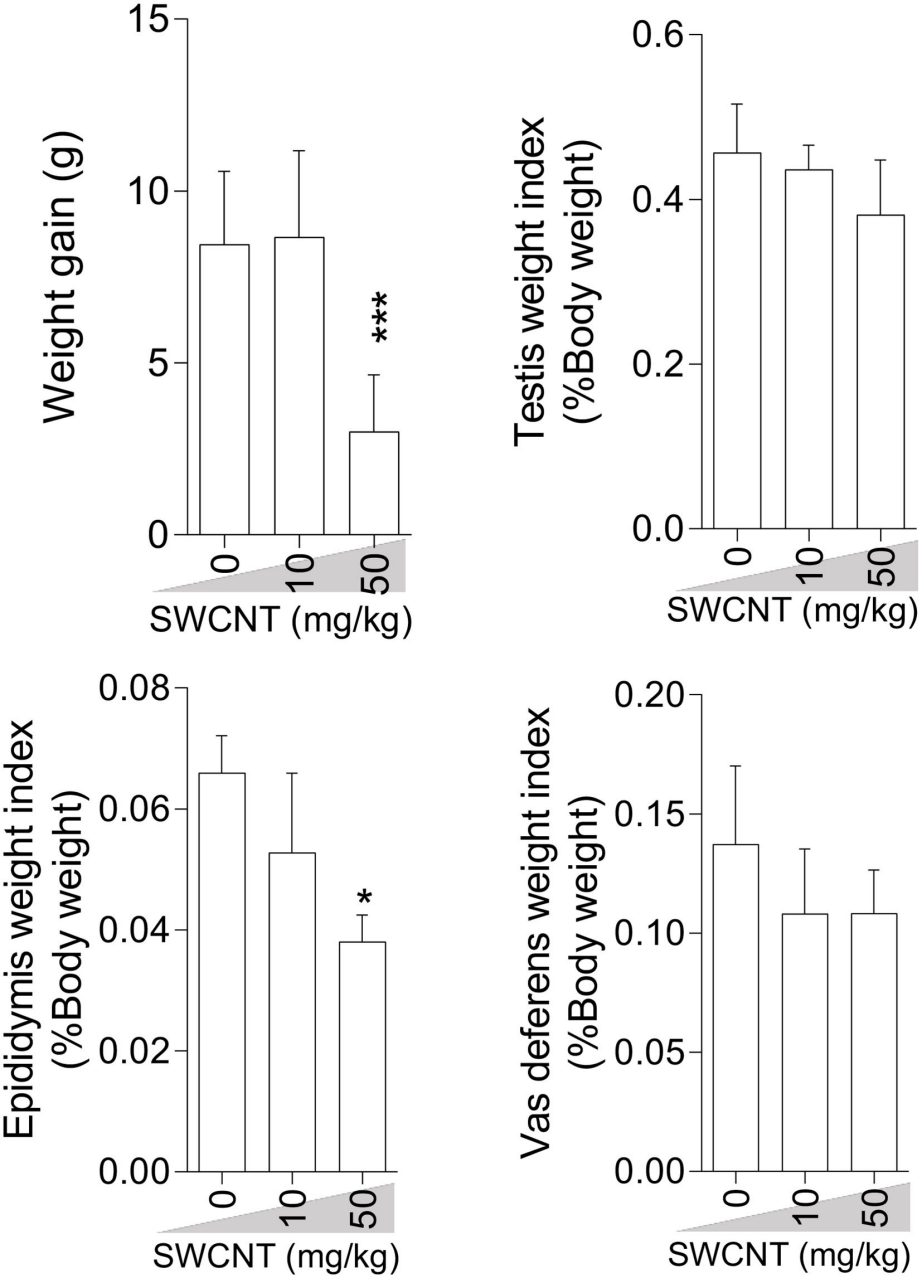
Effect of single-walled carbon nanotubes (SWCNT) on body weight gain and reproductive organ weight index in mice (mean ± SEM, *n* = 6). Asterisks indicate significantly different from the control (0 ppm) group (*P < 0.05, ***P < 0.001).

**Figure 2:**
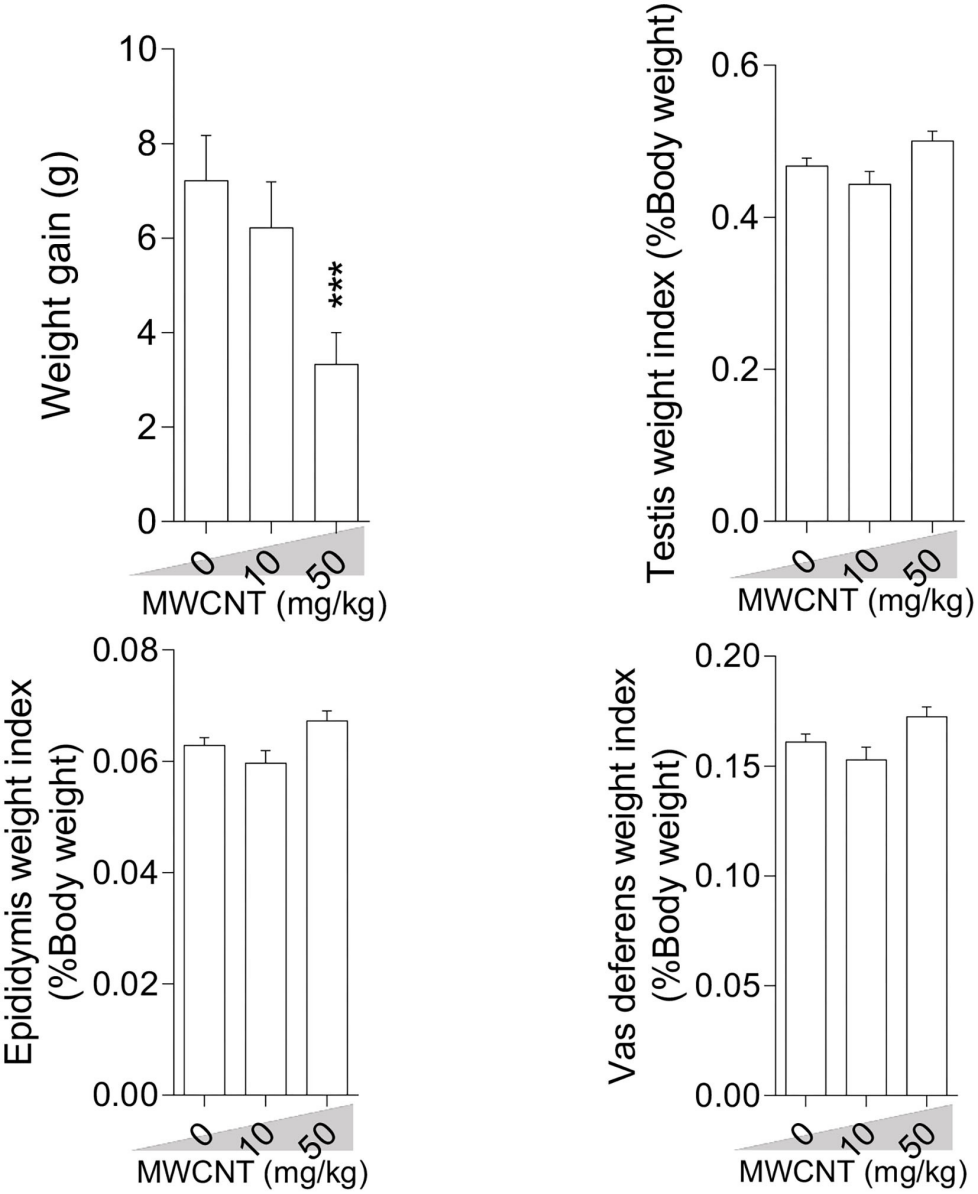
Effect of multi-walled carbon nanotubes (MWCNT) on body weight gain and reproductive organ weight index in mice (mean ± SEM, *n* = 6). Asterisks indicate significant differences from the control group (***P < 0.001).

Bodyweight gain was significantly lower in mice treated with the high dose (50 mg/kg/day) of SWCNT (Figure 1) and MWCNT (Figure 2). The epididymal weight index was also decreased upon exposure to the high dose SWCNT (Figure 1).

### Epididymal Sperm Attributes

**Figure 3:**
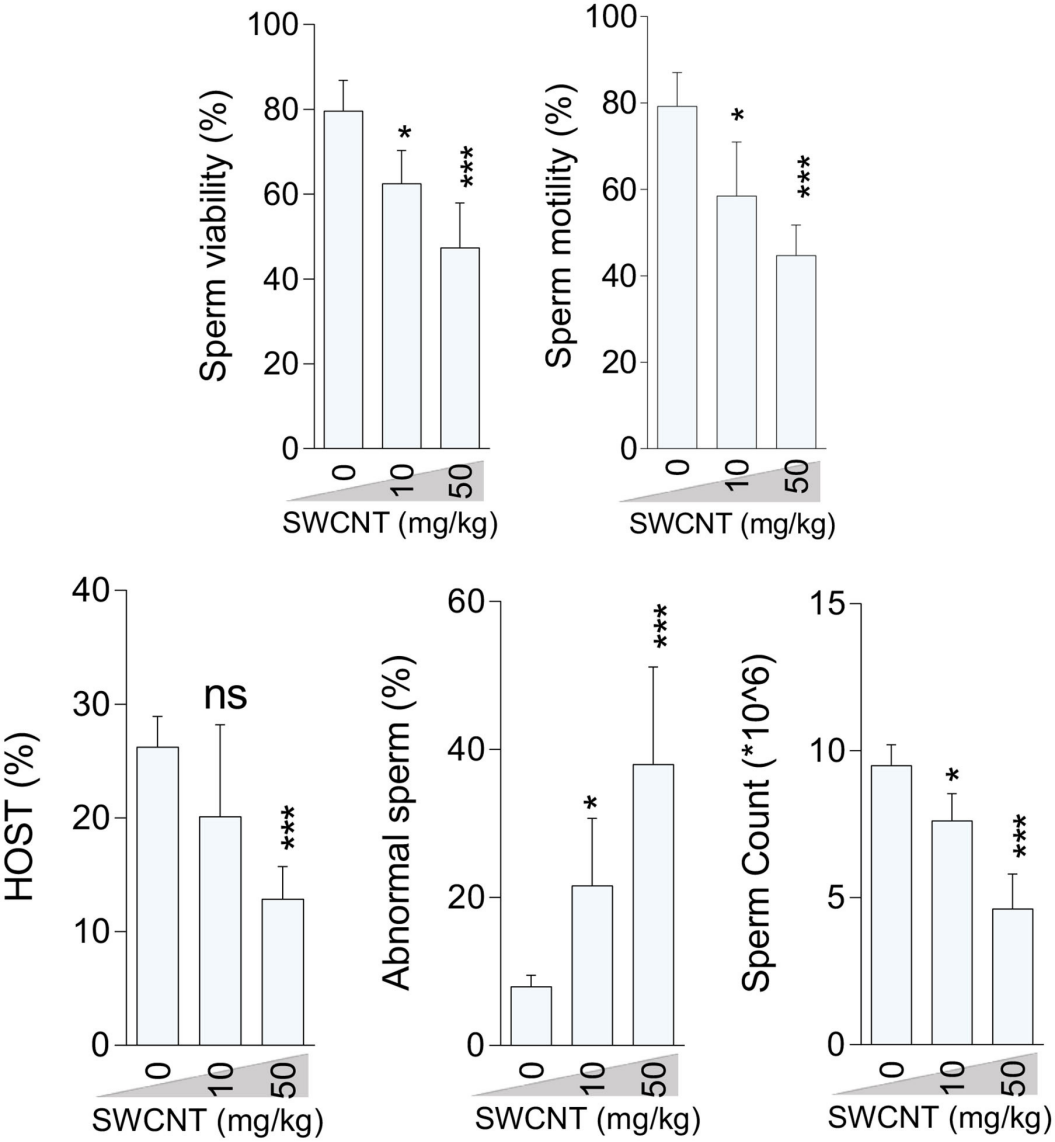
Effect of single-walled carbon nanotubes (SWCNT) on epididymal sperm parameters in mice (mean ± SEM, *n* = 6). HOST: hypo-osmotic swelling test. Asterisks indicate significantly different from the control (0 ppm) group (*P < 0.05, ***P < 0.001).

**Figure 4:**
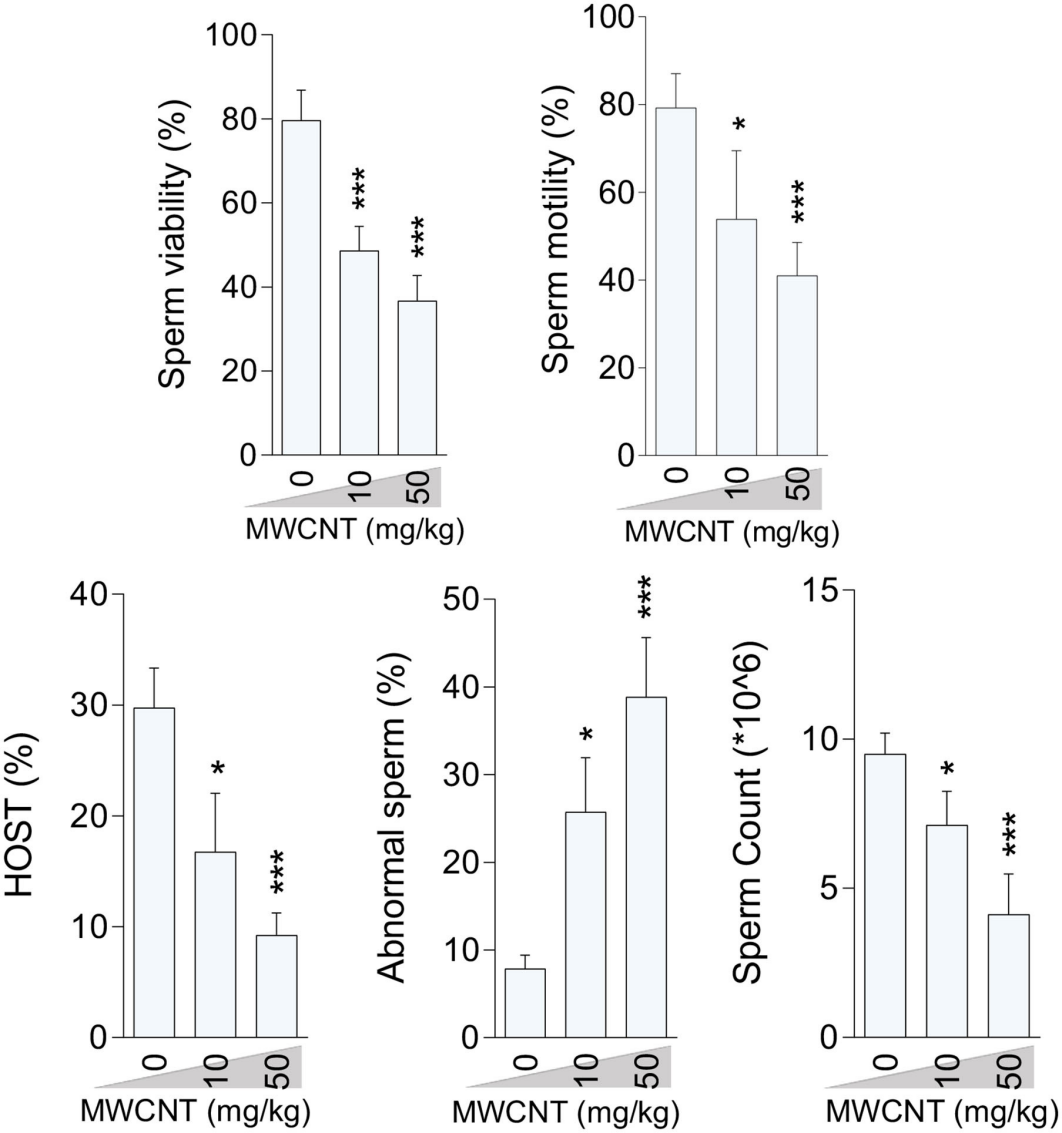
Effect of multi-walled carbon nanotubes (MWCNT) on epididymal sperm parameters in mice (mean ± SEM, *n* = 6). Asterisks indicate significantly different from the control (0 ppm) group (*P < 0.05, ***P < 0.001).

Epididymal sperm quality decreased due to CNT treatment with more significant deterioration at the high dose for both SWCN (Figure 3) and MWCNT (Figure 4). Briefly, the percentage of sperm viability, progressive motility, and HOST, as well as sperm count, were considerably decreased in the animals exposed to 10 and 50 mg/kg of SWCNT (Figure 3) and MWCNT (Figure 4). However, the percentage of sperm abnormality was increased in both CNTs- exposed mice.

### Oxidative Stress (OS) Indices

**Figure 5:**
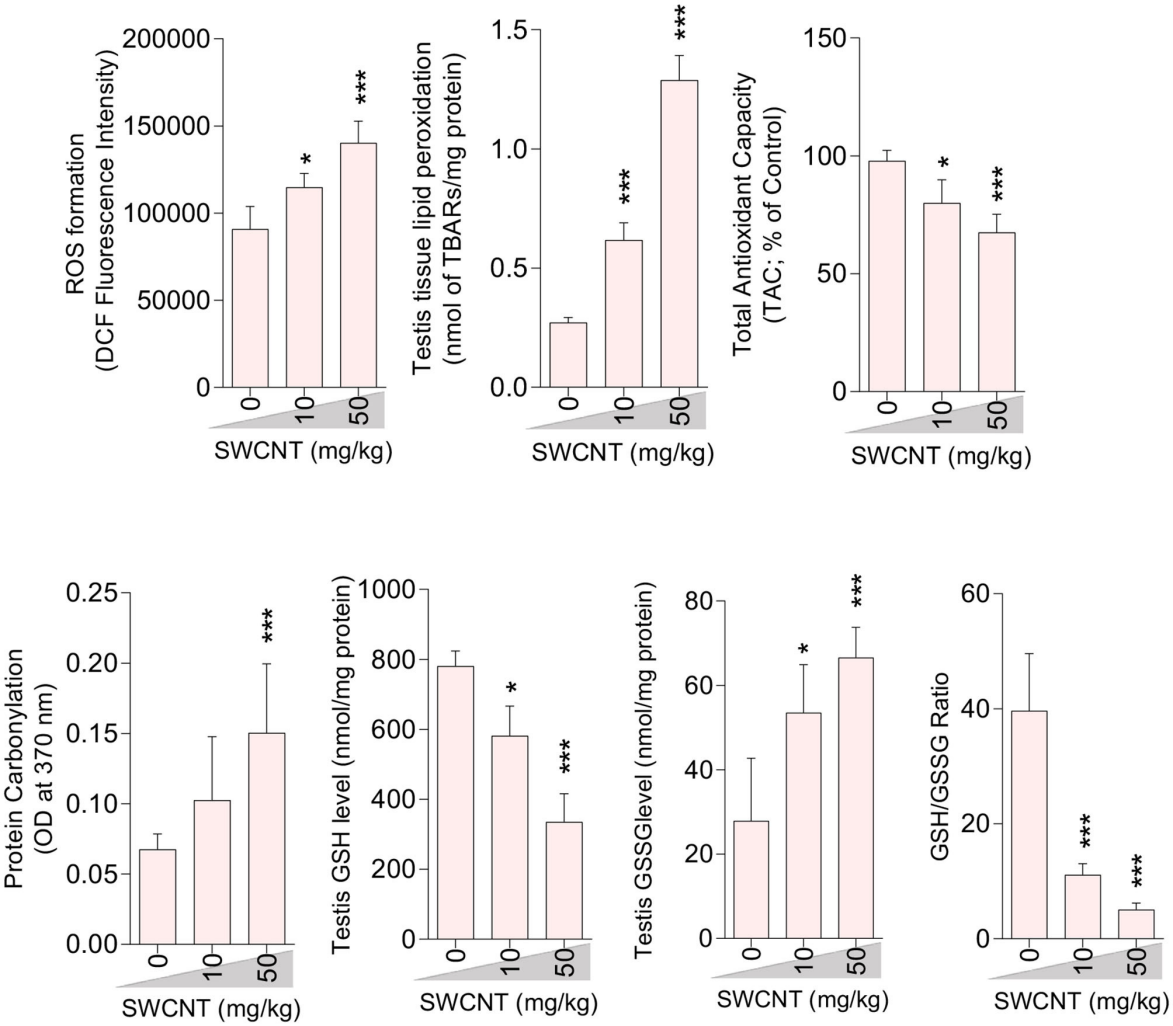
Effect of single-walled carbon nanotubes (SWCNT) on oxidative stress parameters in the testis of mice (mean ± SEM, *n* = 6). Asterisks indicate significantly different from the control (0 ppm) group (*P < 0.05, ***P < 0.001).

**Figure 6:**
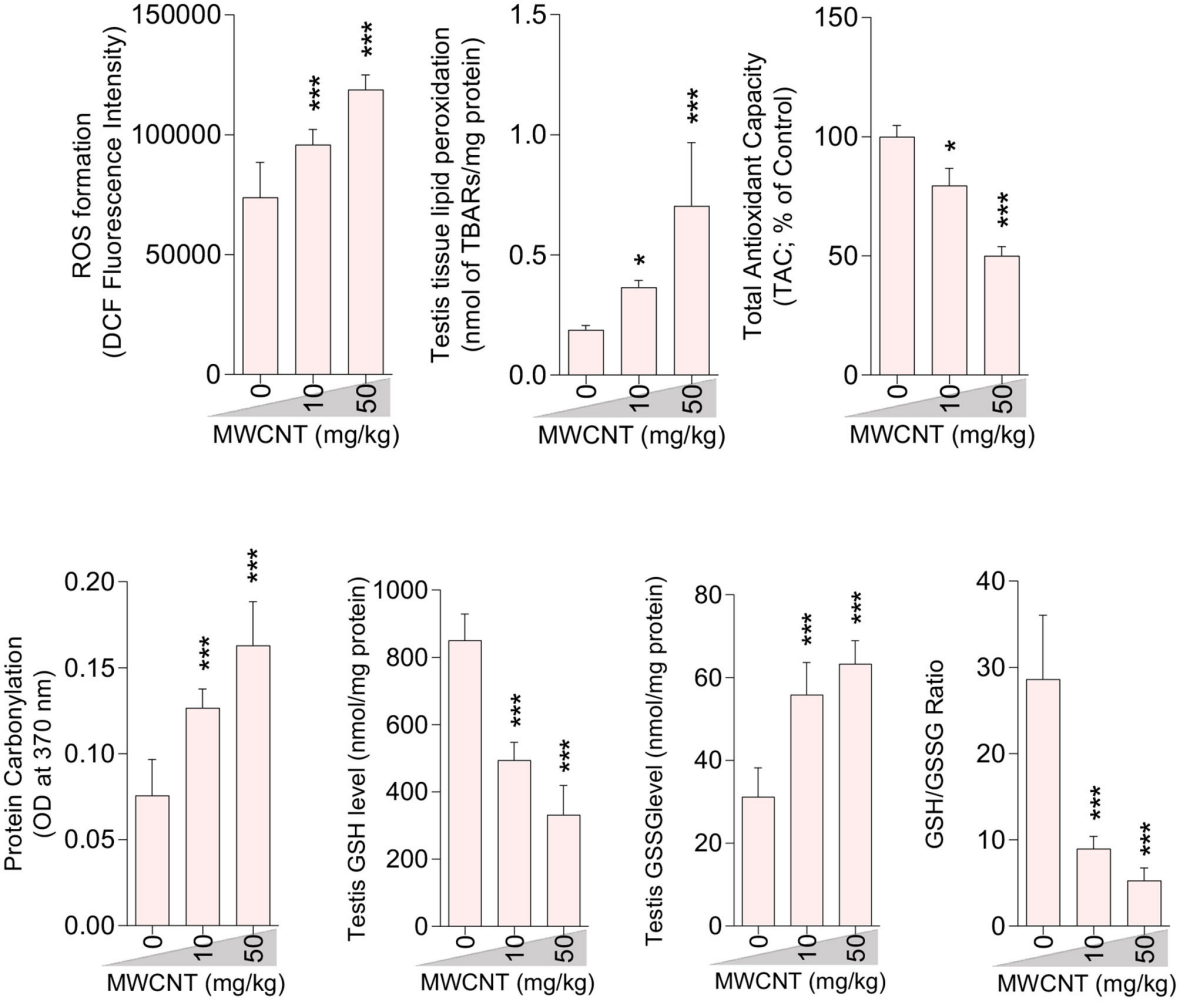
Effect of multi-walled carbon nanotubes (MWCNT) on oxidative stress parameters in the testis tissue of mice (mean ± SEM, *n* = 6). Asterisks indicate significantly different from the control (0 ppm) group (*P < 0.05, ***P < 0.001).

A significant decrement in total antioxidant capacity and GSH/GSSG ratio, along with an increase in ROS formation, TBARS content, GSSG level, and protein carbonylation, were recorded in the testis of mice treated with higher doses of CNTs. Testicular GSH levels were also considerably decreased in both CNT groups (Figures 5, 6).

**Figure 7:**
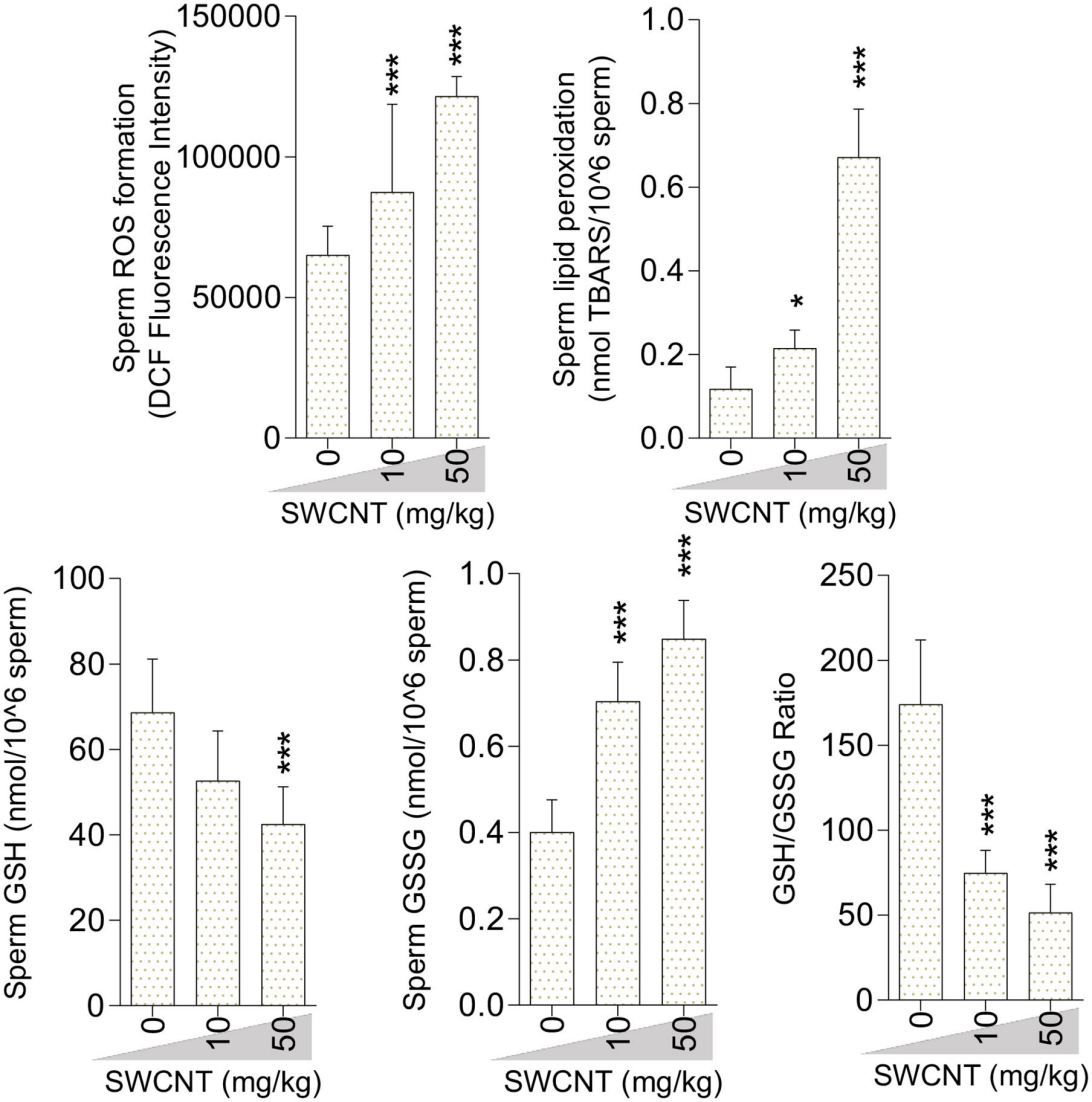
Oxidative stress biomarkers in the sperm isolated from single-walled carbon nanotubes (SWCNT)-treated animals (mean ± SEM, *n* = 6). Asterisks indicate significantly different from the control (0 ppm) group (*P < 0.05, ***P < 0.001).

**Figure 8:**
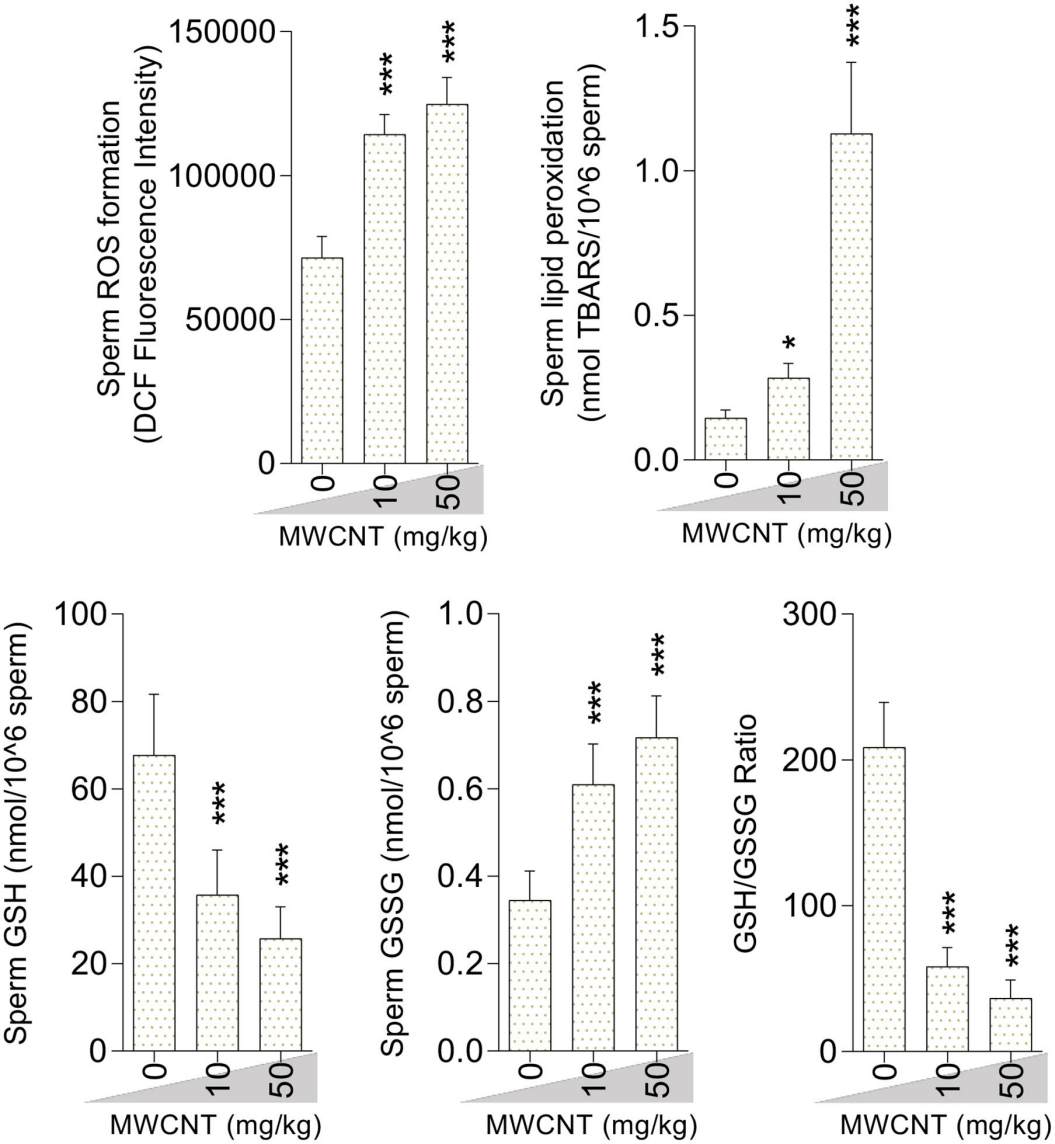
Effect of multi-walled carbon nanotubes (MWCNT) on oxidative stress biomarkers in mice sperm (mean ± SEM, *n* = 6). Asterisks indicate significantly different from the control (0 ppm) group (*P < 0.05, ***P < 0.001).

Sperm ROS formation, TBARS content, and oxidized glutathione levels were notably increased in the mice treated with SWCNT (Figure 7) and MWCN (Figure 8). Conversely, the reduced glutathione level and the ratio of GSH/GSSG decreased in both CNT groups (Figures 7, 8).

### Mitochondrial Parameters of Epididymal Spermatozoa

**Figure 9:**
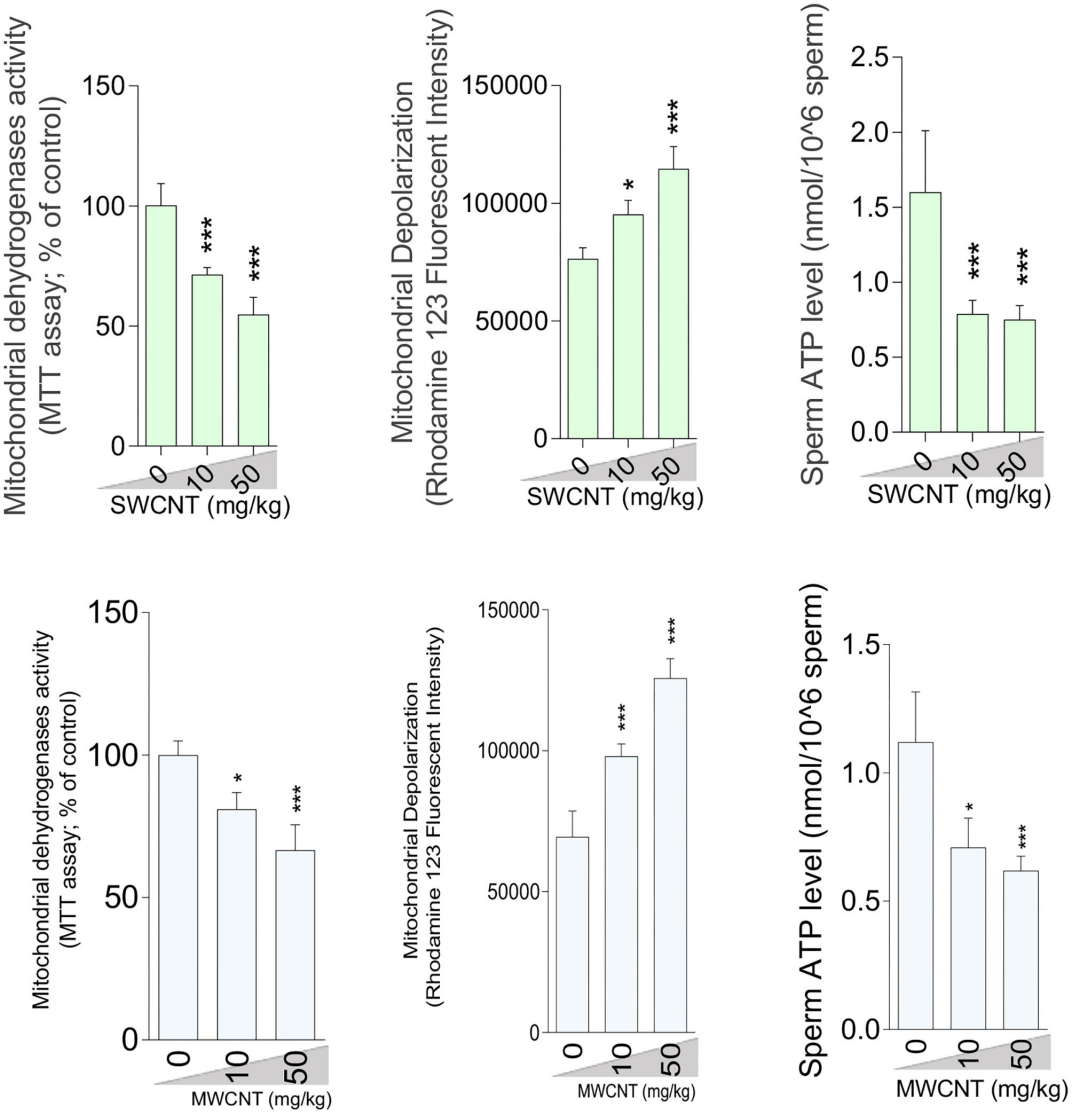
Sperm mitochondrial impairment in carbon nanotubes-exposed animals. (mean ± SEM, *n* = 6). Asterisks indicate significantly different from the control (0 ppm) group (*P < 0.05, ***P < 0.001).

A considerable increment in mitochondrial depolarization and significant decrements in dehydrogenase activity and ATP content were recorded in the isolated epididymal sperm at both doses of SWCNT and MWCN (Figure 9).

### Histopathological and Stereological Alterations in the Testis

**Figure 10:**
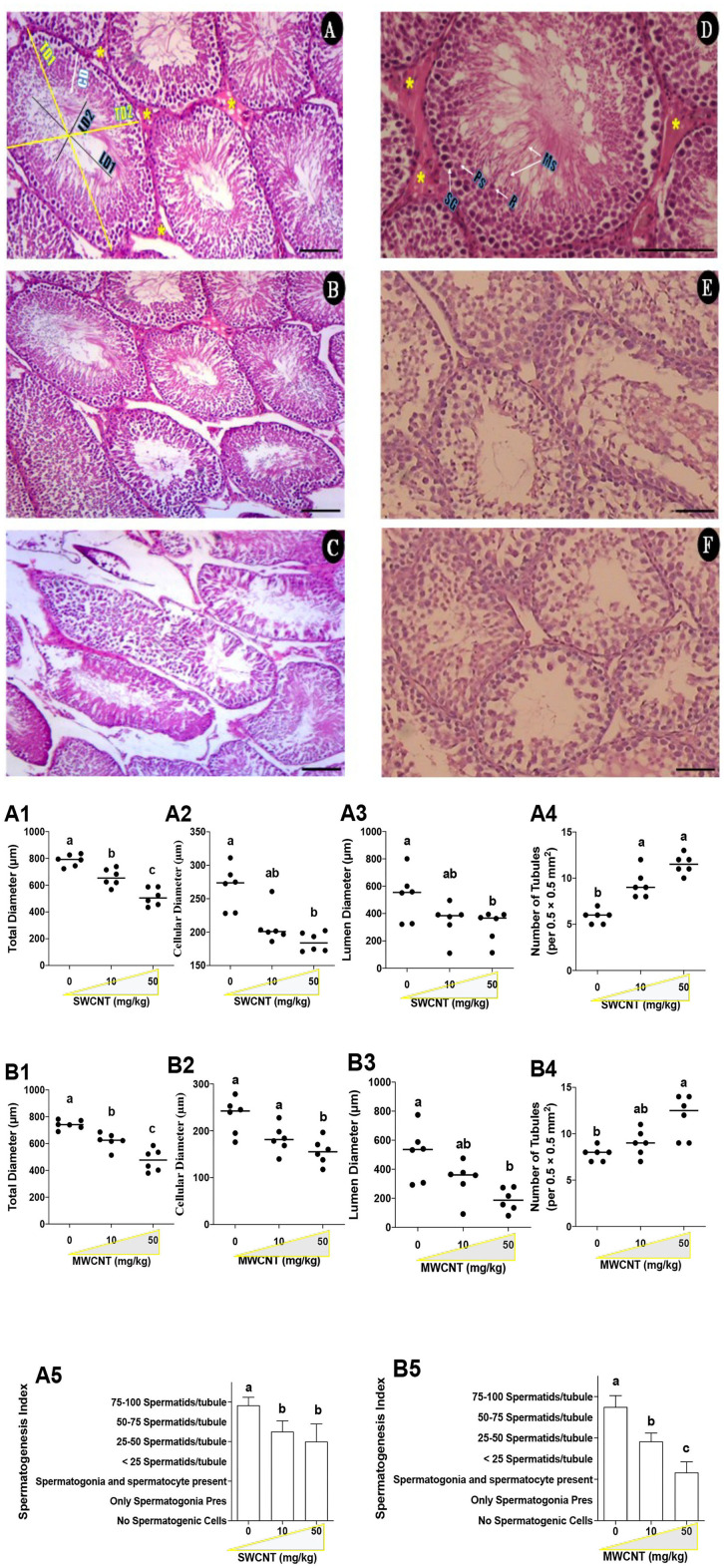
Histopathological changes of the testis in carbon nanotubes-exposed mice. (**A–C**; Control and SWCNT): seminiferous tubules (ST) in control **(A)**, low **(B)**, and high **(C)** SWCNT dose. Total diameter (TD1-2), lumen diameter (LD1-2), cellular diameter (CD); Leydig cells (LCs) clusters (*). (**D–F**; Control and MWCNT; **E**: low dose and **F**: High dose): In the control group **(D)**, the seminiferous epithelium consists of maintaining compartments, spermatogonia (SG), primary spermatocytes (Ps), round spermatids (R), and maturing spermatozoids (Ms), as well as LCs (*). H and E staining; Magnification: ×20 & 40; Scale bar: 100 μm. SWCNT: single-walled carbon nanotubes and MWCNT: multi-walled carbon nanotubes. **(A,B)**: Stereological indices of seminiferous tubules in SWCNT **(A1–A5)** and MWCNT **(B1–B5)**- treated mice. **(A1,B1)** Total diameters (μm); (**A2,B2)** Cellular diameter (μm); **(A3,B3)** Luminal diameter (μm); **(A4,B4)** Number of seminiferous tubules per unit area (0.5 × 0.5 mm^2^); **(A5,B5)** Spermatogenic index of seminiferous tubules.

Histopathological and stereological changes in the testis are shown in Table 1 and Figure 10. Seminiferous tubule (ST) injury and tubules' desquamation significantly increased in SWCNT- and MWCNT- challenged mice. Stereological indices are shown in Figures 10A,B and Table 1. In the mature male mic, the number of STs per unit area of the testis in medium- (10 mg/kg) and high- (50 mg/kg) dose of both CNTs were significantly higher than in the control groups (Figures 10A4,B4). The other stereological indices, including total diameter (Figures 10A1,B1), cellular diameter (Figures 10A2,B2), and lumen diameter (Figures 10A3,B3), were decreased in CNTs (SWCNT and MWCNT) exposed animals (10 and 50 mg/kg) as compared with those in the control group. In the mature male mice exposed to medium- (10 mg/kg) and high- (50 mg/kg) dose of both CNTs, the spermatogenic index was considerably decreased as compared with the control groups (Table 1 and Figures 10A5,B5). However, the minimum index was recorded in the group challenged with the high dose of MWCNT (Figure 10B5 and Table 1).

**Table 1:** Histopathological alterations in CNTs-treated mice.

	**Tubular** ** injury**	**Tubular** ** desquamation**	**Spermatogenic** ** index**
Control (vehicle-treated)	–	–	1
SWCNT, 10 mg/kg	+	++	0.9
SWCNT, 50 mg/kg	++	+++	0.7
MWCNT, 10 mg/kg	++	+++	0.7
MWCNT, 50 mg/kg	++	+++	0.6

*+, Mild; ++, Moderate; +++, Severe histopathological alterations; CNTs, carbon nanotubes; SWCNT, single-walled carbon nanotubes; MWCNT, multi-walled carbon nanotubes*.

It is noteworthy to mention that we found no significant changes in most parameters assessed in the current study when two kinds of nanotubes were compared.

### Fertility Rate

In the SWCNT-challenged mice, the high dose-treated males (50 mg/kg) sired smaller litter size (LS, 7.1 ± 0.51) as compared with that in the low (10 mg/kg) -treated mice (11.5 ± 0.32) and control (12.5 ± 2.01) mice (*p* < 0.05); nevertheless, there were no considerable differences between the low and control mice. However, there was the same trend between SWCNT and MWCNT exposed animals where in MWCNT- treated mice, LS was 6.8 ± 0.21 in the high dose and 10.9 ± 1.05 in the low- treated animals.

## Discussion

Despite plentiful applications for carbon nanotubes (CNTs) in human lifestyles, such as their use in electronic devices, food packaging, and drug delivery vehicles, the likely impact of their increased uptake by living systems has not been sufficiently studied. Recently, CNTs have received considerable attention in biomedicine and biological fields, and concerns have been raised about these materials' hazardous effects on biological systems. Indeed, a part of CNT-induced toxicity might be related to the impurities presented to these compounds (73). Metals (e.g., iron, yttrium, nickel, molybdenum, and cobalt) are used as catalysts in CNTs synthesis. It has been reported that it is impossible to entirely remove the catalyst metals from CNTs' structures (74). The SWCNT and MWCNT used in the present investigation were obtained from the US Research Nanomaterials Inc. (Houston, TX 77084, USA). Based on the level of components (Supplementary Tables 1, 2), metals' concentration in these products seems not to be toxic. However, according to the current work's findings, the deleterious impact of the metallic impurities in CNTs on the reproductive system could not be ruled out. Hence, the role of carbon-based compounds on mammalian reproductive-related intracellular parameters is still incompletely understood.

Utilization of nanomaterials such as CNTs has dramatically increased, especially in the field of biomedicine and drug delivery systems; therefore, elucidation of the mechanism(s) of their adverse effects is essential for the rational use of these compounds. In the current study, we examined the detrimental effects of CNTs on the male reproductive system, with emphasis on several intracellular organelles and functional indices. It was found that exposure to CNTs adversely impacted the male reproductive system and function. Oxidative stress (OS) and mitochondrial dysfunctionality seem to be the primary mechanisms in inducing CNT toxicity in the reproductive system. Testicular germ cells are protected via BTB (75). Bai et al. (45) showed that continuous intravenous (i.v) injection of CNTs to BALB/c mice resulted in reversible injury to the testes due to testicular CNT accumulation and OS, even though fertility was not affected by CNT treatment.

There is a good body of evidence demonstrating any alteration in internal organs, and body weight (BW) may be a sensitive index of xenobiotics' undesired effects (76). A considerable decrement in the relative weight of epididymis and BW of mice receiving a high dose of SWCNT (50 mg/kg) for 35 days is an indication of induced epididymal atrophy. In line with our observations, Olugbodi et al. (77) and Watanabe (78) reported reductions in the relative weight of epididymis and testes (after 7 days) and vesicular and prostate glands (after 19 days) following nanoparticle administration to rats. We found the toxic effects of high CNTs doses on body weight and epididymal weight. The lower BW might have been caused by the decreased weight of the accessory sex organs and perhaps other reproductive and non-reproductive tissues that were not studied in this investigation. However, the lower BW showed that the overall health condition of the male mice exposed to the high dose of CNTs was adversely impacted. The normal growth and function of accessory sex organs are crucial for male fertility success (79, 80). The detrimental effects of toxic elements, xenobiotics (i.e., CNTs) on BW of the laboratory animal species is well-established in the literature (81, 82). The recorded decrements in BW of mice in this investigation could also be ascribed to changes in physiological processes that affect the feeding behavior, resulting in decreased food intake and the BW. Depend on the experimental conditions, daily or weekly body weight gain during the test period, absolute weight alterations of other male accessory sex gland (i.e., seminal vesicle, prostate glands), and feeding behavior (food consumption rate) was not determined in the current study; hence, the presence of any correlation between these indices with CNTs-induces adverse effects warrants further investigations. Previous reports on the effect of CNTs on BW and internal organs are contradictory. For instance, De Jong et al. (83) reported considerable growth retardation in rats receiving silver nanoparticles (i.v) for 28 days, while Zhang et al. (84) showed that injection of nanoparticles for about 2 weeks caused temporary and reversible alterations in mice BW. Our data contradict the findings of Lee et al. (85), who reported no significant dose-related BW changes upon exposure of rats to silver and gold nanoparticles (10 and 100 μg/kg/day). Bai et al. also reported that administration of MWCNTs (5 mg/kg, size 100 nm, single-dose, i.p, at different time intervals) had no significant effects on male fertility in mice (45). Such discrepancies might be ascribed to the CNTs type, dose, route of administration, and particulate size of the CNTs, as well as the animal species used in various experiments. On the other hand, the increased ROS level, TBARS content, protein carbonylation, and oxidized glutathione, along with decreased glutathione level (reduced form) and total antioxidant capacity in the testis and sperm, indicated that the mice administered with 10 or 50 mg/kg/day of SWCNT and MWCNT for 35 days were under some kinds of stress which could affect their well-being and weight.

The nanoparticles enter the treated animals' circulatory system mainly as particulate matter; accordingly, interacting with blood constituents to induce coagulative reactions (85). Olugbodi et al. (77) reported significant decreases in the red blood cells (RBC) and hemoglobin (Hb) levels in rats exposed to 10 and 50 mg/kg BW of silver nanoparticles for 1 week, reflecting the hematotoxic effects of CNTs. This phenomenon might be ascribed to the OS-generated ROS that compromises the membrane integrity (86). Hence, more investigations are needed to evaluate this phenomenon in the paternal and filial generations exposed to SWCNT and MWCNT.

As mentioned, the functionality of BTB, BBB, and HTB might be interrupted by CNTs. CNTs can pass-through these barriers and directly affect the reproductive-related organs, spermatogenesis, and neuroendocrine-related pathways. Multiple investigations also showed that CNTs could cross BTB, placental, and BBB (87). Hence, the accumulation of CNTs in the testis may interrupt Leydig and Sertoli cells' functionality with a wide range of abnormalities in steroidogenesis and germ cell differentiation. It has been reported that CNTs could affect the serum level of sex hormones (82, 88). Therefore, assessing serum or tissue level of endocrine hormones might also provide valuable data about CNTs' reproductive toxicity mechanisms.

On the other hand, interestingly, it has been reported that CNTs could reduce the side effects of endocrine disruptors (e.g., Phthalates and Triclosan) (89, 90). The recent impacts of CNTs could be attributed to decreasing the bioavailability of endocrine disruptors (89, 90). Therefore, further researches are needed to assess the effect of prenatal exposure to SWCNT and MWCNT on the development of the offspring. Exposure to CNTs induced notable changes in the reproductive system; however, the male's fertilizing capacity is the only parameter expected to affect the population dynamics. To this end, we studied the impact of CNTs on the sperm quantitive and qualitative indices related to fertility. Both CNTs adversely impacted epididymal sperm quality. Our data contrasted with Ivanov et al.'s findings who did not observe any *in-vitro* cytotoxic effect of MWCNT; additionally, they showed a considerable improvement in the cell's activity via their entire lifespan (91). These discrepancies might be due to the type of experimental models (*in-vitro* vs. *in-vivo*) and the treatment doses of CNTs. Wiwanitkit et al. (92) reported that nanoparticles accumulated in the sperm tail and head, causing 25% immobility in male gametes (92). In line with our observations, it was reported that nanoparticles could change testicular morphology (93). Sperm motility, as a potential index of fertilizing ability, can adversely be affected by environmental (94, 95), industrial (96, 97), and pharmaceutical agents (98). In line with previous researches (20, 21, 26–31, 57), we also observed a significant decrement in sperm viability, forward motility, plasma membrane integrity (HOS test), and sperm count, as well as an increment in sperm abnormality following treatment with CNTs. Severe OS along with mitochondrial dysfunction would impair the spermatogenesis and fertility through decrements in sperm viability, count, motility, and steroidogenesis, and an increment in the percentage of abnormal sperm, as well as alterations in histomorphology of the testes or accessory sex organs (20, 26–28, 30). It has been reported that the size and functional groups of carbon-based nanomaterials play pivotal roles in determining their toxic roles on the male germ cells (94, 99).

The indices of OS were dramatically intensified in the testis and sperm of CNT-treated mice. It is known that gametogenesis is very sensitive to the OS (30, 34), which has deleterious effects on bio-membranes, mitochondria, and other macromolecules (100). Mitochondrial impairment due to OS induction by CNTs in the present experiment could be an essential factor in CNT- induced reproductive toxicity. There are ample data in the literature showing that the abnormal levels of cellular glutathione (oxidized/reduced GSH) are closely interconnected with the induction of OS in reproductive organs (20, 21, 27, 29); therefore, CNT-induced decreases in reduced GSH levels, and increases in oxidized GSH level in male gonads and gametes could trigger severe OS and complications. Spermatozoa are very sensitive to peroxidation due to the high level of polyunsaturated fatty acids (PUFAs) in the plasma membrane (26, 32), adversely affecting the fertility potential (26, 33, 34). On the other hand, male gametes' antioxidant capacity is considerably low; hence, the enzymatic and non-enzymatic antioxidant systems in seminal plasma are required for sperm protection via free radical scavenging (101).

Exposure to SWCNT and MWCNT caused severe OS (102), inflammation, and histopathological alterations in various organs and cells, including the lungs (103), kidneys (15), liver (104), human skin HaCat cells (105), melanoma cells (106), lung cells (107), human microvascular endothelial cells (108), human embryonic kidney (HEK293) cells (15), human neuroblastoma cells (109), human mesothelial cells (110), and human keratinocytes (42). The increased contents of reactive oxygen species (ROS) and thiobarbituric acid reactive substance (TBARS), severe disruption in biomembranes, and disturbed mitochondrial redox state are well-described events involved in the mechanisms of CNT-mediated cytotoxicity (14, 105, 108, 111). However, a few studies have focused on the OS-induced male subfertility in animals exposed to CNTs (45, 87, 94). Based on the above references, it is likely that OS and its associated events might be involved in the CNT-induced reproductive toxicity in males. Altogether, the observed adverse effects of CNTs on the reproductive indices, including histopathological and stereological indices, mitochondrial and sperm characteristics as well as alterations in OS parameters could be attributed to the intensified peroxidation of PUFAs, decreased total antioxidant capacity (FRAP test), and depleted GSH contents in the gonad and gametes of the mice.

There is a close relationship between energy production in mitochondria and the functionality of the male reproductive cells (112). Thus, having healthy mitochondria guarantees the proper function of the reproductive cells. In this study, both CNTs caused a collapse in mitochondrial membrane potential and decreased mitochondrial dehydrogenase activities and ATP content. These mitochondrial alterations point to a tremendous ROS generation via mitochondrial electron transport chain and increases in the permeability of transition pores (113, 114). However, clarification of the precise mechanism of SWCNT and MWCNT action on HPG-S mitochondria in perinatal periods and filial generations requires further studies.

## Conclusions

This investigation is the first report of the intracellular toxicity of two crucial CNTs, SWCNT and MWCNT, on mammalian testis and gametes. Based on our observation, it was found that CNTs-associated subfertility could be induced directly via dysfunctional male gonads and gametes and/or might be affected through alterations in the functionality of the hypothalamus-pituitary axis. In the current study, we almost found a similar trend on testis and sperm parameters related to oxidative stress and mitochondrial functionality in CNTs-treated groups; however, contradictory results in relation to weight gains, testes, epididymides, and vas deferens weight index were observed between the groups exposed to SWCNT and MWCNT. Hence, despite the more toxic effects of SWCNT on the body- and reproductive organs- weight, further investigations are needed to elucidate the precise cellular-related pathways associated with the reprotoxicity of these CNTs, such as apoptosis, autophagy, and inflammatory-related routes. Exposure of mice to these nanomaterials resulted in considerable alterations in male reproductive health via OS-induced mitochondrial dysfunction through gonadal injury or gametic abnormality. The induction of OS is of concern because it is likely that these CNTs might have detrimental effects on males' fertility at higher doses over a more extended period. These observations suggest the need for further studies to clarify the effects of these substances on fertilization and zygote development in male and female F1 and F2 generations by evaluating intracellular-related routes such as inflammatory responses and apoptosis and autophagic pathways.

## Data Availability Statement

The raw data supporting the conclusions of this article will be made available by the authors, without undue reservation.

## Ethics Statement

Animal handling and experimentation procedures were approved by the Experimental Animal Welfare and Ethics Committee of Shiraz University of Medical Sciences, Shiraz, Iran (95-01-36-11290).

## Author Contributions

OF: methodology and investigation. RH: methodology, software, and validation. MJZ: writing—original draft and validation. SR-M: methodology and validation. MK: performed the research. ME: investigation. AJ: supervision, methodology, and validation. MMO: conceptualization, investigation, analyzed the data, supervision, and writing—original draft. All authors contributed to the article and approved the submitted version.

## Conflict of Interest

The authors declare that the research was conducted in the absence of any commercial or financial relationships that could be construed as a potential conflict of interest.
