# Supplementary material for: Spermatotoxic Effects of Single-Walled and Multi-Walled Carbon Nanotubes on Male Mice
Source: Front Vet Sci. 2020 Dec 17;7:591558. doi: 10.3389/fvets.2020.591558 (PMC7775657; doi:10.3389/fvets.2020.591558)
Supplement: Supplementary file 1 [file Table_1.docx]

**For more details please check: www.us-nano.com**

| **Supplementary Table 1. SWCNTs Properties:**  **Analysis Method, Energy Dispersive X-ray Spectroscopy** | |
| --- | --- |
| **Determined Components** | **Level (%)** |
| C | 96.30 |
| Al | 0.08 |
| Cl | 0.41 |
| Co | 2.91 |
| S | 0.29 |
| **Physical Properties** | |
| Young’s Modulus (GPa) | 1054 |
| Tensile Strength (GPa) | 150 |
| Thermal Conductivity (W/m.K) | 3000 |
| Electrical Conductivity (S/m) | 10^5^-10^7^ |
| SWCNTs: Single-walled carbon nanotube; C: Carbon; Al: Aluminum; Cl: Chlorine, Co: Cobalt; S: Sulphur. -COOH Content：2.73 wt%; SWCNT-OH (Stock # US4112). | |
