# Supplementary material for: Spermatotoxic Effects of Single-Walled and Multi-Walled Carbon Nanotubes on Male Mice
Source: Front Vet Sci. 2020 Dec 17;7:591558. doi: 10.3389/fvets.2020.591558 (PMC7775657; doi:10.3389/fvets.2020.591558)
Supplement: Supplementary file 2 [file Table_2.docx]

| **Supplementary Table 2. MWCNTs Properties:**  **Analysis Method, Energy Dispersive X-ray Spectroscopy** | |
| --- | --- |
| Determined Components | Level (%) |
| C | 98.39 |
| Cl | 0.45 |
| Fe | 0.23 |
| Ni | 0.93 |
| **Physical Properties** | |
| Young’s Modulus (GPa) | 1200 |
| Tensile Strength (GPa) | 150 |
| Density (g/cm^3^) | 2.6 |
| Thermal Conductivity (W/m.K) | 3000 |
| Electrical Conductivity (S/m) | 10^5^-10^7^ |
| MWCNTs: Multi-walled nanotubes; C: Carbon; Cl: Chlorine, Fe: Iron; Ni: Nickle. MWNTs > 95 wt %; -OH Content： 1.76 wt％; MWCNT-OH (Stock # US4310), 20-30 nm. | |

**For more details please check: www.us-nano.com**
